# The interactions of metal cations and oxyanions with protein tyrosine phosphatase 1B

**DOI:** 10.1007/s10534-017-0019-9

**Published:** 2017-05-24

**Authors:** Kshetrimayum Birla Singh, Wolfgang Maret

**Affiliations:** 1 Department of Zoology, Pachhunga University College, Mizoram University, Aizawl, 796001 Mizoram India; 2 Metal Metabolism Group, Department of Biochemistry, Division of Diabetes and Nutritional Sciences, Faculty of Life Sciences and Medicine, King’s College London, Franklin-Wilkins Building, 150 Stamford Street, London, SE1 9NH UK

**Keywords:** Metal cations, Oxyanions, Inhibition, Protein tyrosine phosphatase

## Abstract

Protein tyrosine phosphatases are not considered to be metalloenzymes. Yet, they are inhibited by zinc cations and metal and non-metal oxyanions that are chemical analogues of phosphate, e.g. vanadate. Metal inhibition is generally not recognized as these enzymes are purified, supplied, and assayed with buffers containing chelating and reducing agents. We screened a series of cations and anions for their capacity to inhibit protein tyrosine phosphatase 1B and discuss the ensuing general issues with inhibition constants reported in the scientific literature. In contrast to zinc, which binds to the phosphocysteine intermediate in the closed conformation of protein tyrosine phosphatase 1B when the catalytic aspartate has moved into the active site, other divalent cations such as cadmium and copper may also bind to the enzyme in the open conformation. Inhibition by both anions and cations, conditions such as pH, the presence of metal ligands such as glutathione, and the existence of multiple conformational states of protein tyrosine phosphatases in the reaction cycle establish a complex pattern of inhibition of these important regulatory enzymes with implications for the physiology, pharmacology and toxicology of metal ions.

## Introduction

Protein tyrosine phosphatases, a family of 107 enzymes in humans, are key regulators of cellular phosphorylation signalling (Tonks 2013). To fulfil this role, they are regulated themselves by a variety of processes, e.g. covalent modification (phosphorylation, sumoylation), dimerization and a remarkable redox chemistry involving the catalytic cysteine residue. The enzymes work with two catalytic steps. The phosphorylated substrate binds and transfers its phosphate group to the catalytic cysteine to form a phosphocysteine intermediate and the dephosphorylated susbstrate, the first product. In the second, rate-limiting step, the enzyme is dephosphorylated forming phosphate, the second product. Inhibition of protein tyrosine phosphatase 1B (PTP1B, PTPN1) by zinc ions was noticed over 35 years ago (Brautigan et al. 1981). It gains significance only now because recent developments in the field of zinc biology provide evidence for zinc ions serving functions as cellular signalling ions in a range of concentrations that is commensurate with the affinity of PTP1B and for zinc (Haase and Maret 2003; Krezel and Maret 2008). Protein tyrosine phosphatases are thought to be targets of these signalling zinc ions, adding yet another layer of regulation to these important enzymes. Zinc inhibition was found to be exquisitely tight. A *K*_i_ value of 21 pM was determined for receptor protein tyrosine phosphatase beta (RPTPβ) (Wilson et al. 2012). This affinity is close to that of genuine metalloenzymes for zinc. Thus, in contrast to zinc enzymes that are activated and employ their metal for catalysis, other enzymes are inhibited by zinc and zinc needs to be removed from the enzymes for them to become active (Maret 2013). Such a mode of action seems to have been generally overlooked as active enzymes are prepared, supplied, and assayed in the presence of chelating agents that bind the inhibitory metal ion and hence mask the inhibition. Specificity in the regulation of PTPs was noted when zinc inhibition was observed in the closed conformation of PTP1B (Bellomo et al. 2014). Protein ligands for binding the inhibitory metal ion become available only in the closed conformation. They are thought to involve the carboxylate group of the catalytic aspartate (Asp181) and the phosphate group of the phosphocysteine intermediate. This mode of inhibition is different from the redox modulation of PTP activity, which occurs in the open protein conformation when the catalytic cysteine is not modified. In addition to metal oxyanions, non-metal oxyanions such as nitrate bind to the catalytic site of PTP1B (Kenny et al. 2014). In order to determine the relative inhibition under the same conditions, we screened the inhibitory capacity of a range of cations and anions with a versatile and sensitive fluorimetric enzymatic assay (Bellomo et al. 2014). The results indicate that inhibition requires the binding of an anion first to make possible the interaction of the enzyme with a cation. Such an inhibition has wide implications for the physiology, pharmacology, and toxicology of metal ions.

## Experimental procedures

### Reagents

Molecular biology-grade HEPES, tris(2-carboxyethyl)phosphine hydrochloride (TCEP), nitrilotriacetic acid (NTA), zinc chloride (ZnCl_2_), copper sulphate (CuSO_4_), cadmium chloride (CdCl_2_), lead nitrate (Pb(NO_3_)_2_), ferric chloride (FeCl_3_), ammonium iron(II) sulphate [(NH_4_)_2_Fe(SO_4_)_2_·6H_2_O], chromium chloride (CrCl_3_), lanthanum chloride (LaCl_3_), lithium chloride (LiCl), manganese chloride (MnCl_2_), nickel chloride (NiCl_2_), silver nitrate (AgNO_3_), ammonium tetrathiomolybdate [(NH_4_)_2_MoS_4_], sodium tungstate (Na_2_WO_4_), potassium nitrate (KNO_3_), ammonium molybdate [(NH_4_)_2_MoO_4_], sodium arsenate, (Na_2_HAsO_4_·7H_2_O), sodium chromate (Na_2_CrO_4_·4H_2_O), boric acid (H_3_BO_3_), potassium hydrogen carbonate (KHCO_3_), histidine, sodium hydroxide, and Triton X-100 were from Sigma-Aldrich; ammonium heptamolybdate [(NH_4_)_6_Mo_7_O_24_] from BDH Laboratory Supplies, Poole, UK; 6,8-difluoro-4-methylumbelliferyl phosphate (DiFMUP) and 6,8-difluoro-7-hydroxy-4-methylcoumarin (DiFMU) were from Invitrogen. Recombinant human PTP1B, residues 1–299, was from Millipore, supplied in 50 mM Hepes, pH 7.2, 1 mM dithiothreitol (DTT), 1 mM ethylenediaminetetraacetic acid (EDTA), and 0.05% (v/v) NP-40. Chromium (III) histidinate was used because it is the least kinetically inert complex. To prepare chromium (III) histidinate, chromium (III) chloride (20 mmoles) and histidine (60 mmoles) were dissolved in 150 mL water and heated to 80 °C for 15 min, sodium hydroxide (50 mmoles) in 50 mL water was added within four minutes. The blood red complex was used for the experiments.

### Metal cation and oxyanion inhibition assay

Enzymatic activity of PTP1B (2.5 nM final concentration) was assayed fluorimetrically at 25 °C in a buffer containing 50 mM Hepes/Na^+^, pH 7.4, 1 mM NTA, 0.01% (v/v) Triton X-100, freshly prepared 0.1 mM TCEP, and with different concentrations of metal cations or oxyanions. The reaction was initiated by adding the fluorigenic phosphatase substrate DiFMUP. Assays were performed in triplicates in a total volume of 100 µl in 96-well black optical bottom plates (Greiner Bio-One Ltd, Stonehouse, UK). Product formation (hydrolysis of DiFMUP to DiFMU) was monitored at 460 nm emission and 360 nm excitation with a fluorescence plate reader (Synergy HT, BioTek, Winooski, VT). Initial rates were determined from the linear portion of the progress curves. Fluorescence intensity was converted to molar concentrations from measurements with a standard of a DiFMUP–DiFMU mixture.

### Determination of total and free Cu^2+^, Cd^2+^ and Zn^2+^ and their inhibition

In order to determine inhibition constants of the metal cations on the activity of PTP1B, we used Maxchelator (Environmental Research Software, Hallowell, ME) (Bers et al. 2010) to calculate the concentration of free Cu^2+^, Cd^2+^ and Zn^2**+**^ in the buffer (Table 1). The concentrations of EDTA and DDT in the diluted commercial PTP1B used in the assay were incorporated into the calculations for free metal ion concentrations using Maxchelator. NTA (1 mM) in the pH-buffer solutions was used to buffer copper, cadmium and zinc ions. When using 1 mM GSH instead of 1 mM NTA in the assay, the calculations of free zinc ion concentrations were provided by Professor Wojtek Bal (Polish Academy of Science, Warsaw) (Table 2).

**Table 1:** Free metal (II) ion concentrations in solutions metal-buffered with NTA

[Cu^2+^]_total_ (µM)	[Cu^2+^]_free_ (M)	[Cd^2+^]_total_ (µM)	[Cd^2+^]_free_ (M)	[Zn^2+^]_total_ (µM)	[Zn^2+^]_free_ (M)
1	1.18 × 10^−14^	1	2.05 × 10^−11^	10	2.66 × 10^−11^
5	5.95 × 10^−14^	5	1.03 × 10^−10^	50	1.39 × 10^−10^
10	1.19 × 10^−13^	30	3.30 × 10^−10^	200	6.58 × 10^−10^
30	3.66 × 10^−13^	50	1.08 × 10^−9^	300	1.13 × 10^−9^
50	6.23 × 10^−13^	200	5.12 × 10^−9^	700	6.15 × 10^−9^
100	1.31 × 10^−12^	350	1.10 × 10^−8^	800	1.05 × 10^−8^
200	2.96 × 10^−12^	600	3.07 × 10^−8^	900	2.37 × 10^−8^
300	5.07 × 10^−12^	800	8.19 × 10^−8^	950	5.01 × 10^−8^
500	1.18 × 10^−11^	900	1.84 × 10^−7^	970	8.52 × 10^−8^
		980	9.61 × 10^−7^	980	1.29 × 10^−7^
				990	2.57 × 10^−7^

50 mM Hepes, pH 7.4, 1 mM NTA, 0.1 mM TCEP and 0.01% (v/v) Triton X-100. Free metal ion concentrations were calculated using the program MaxChelator

**Table 2:** Free zinc (II) ion concentrations in solutions metal-buffered with glutathione (GSH)

[Zn^2+^]_total_ (µM)	[Zn^2+^]_free_ (M)
1	5.88 × 10^−9^
3	1.76 × 10^−8^
7	4.19 × 10^−8^
10	6.04 × 10^−8^
30	1.92 × 10^−7^
70	5.02 × 10^−7^
100	7.84 × 10^−7^
300	4.57 × 10^−6^
700	4.88 × 10^−5^

50 mM Hepes, pH 7.4, 1 mM GSH and 0.01% (v/v) Triton X-100

The metal buffered solutions were freshly prepared and equilibrated for 15 min before the assay was started by adding enzyme. The reaction was monitored under initial velocity conditions. Inhibition constants (apparent *K*_i_ values) were obtained from fitting semi-logarithmic plots with non-linear regression curves (Sigma plot).

### Zinc analysis by ICP-MS

One of the major issues in determining PTP1B activity is the contamination of zinc in the experimental solutions and buffers. Zinc is a ubiquitous contaminant of laboratory chemicals. In order to monitor zinc levels, total zinc concentrations in solutions and buffers were measured using inductively coupled plasma mass spectrometry (ICP-MS, Perkin Elmer Life Science, model Elan 610 DRC plus). Samples were prepared in 5% (v/v) HNO_3_-washed polypropylene tubes (Elkay, Basingstoke, UK).

## Results

### Effect of metal cations on PTP1B activity

We investigated a total of 20 metal ions (12 cations and 8 oxyanions) with regard to their capacity to modulate the activity of PTP1B (Table 3). Metal cations generally inhibited more strongly than oxyanions. Copper(II), cadmium(II), zinc(II), lead(II) and silver(I) at concentration between 5 and 50 µM, respectively, inhibited between 90 and 99%. Manganese(II) and nickel(II) ions did not inhibit PTP1B at these concentrations. We also investigated the effect of the charge of the cation by employing trivalent (chromium) and monovalent (lithium) cations. They also did not inhibit significantly the activity of PTP1B, confirming previous observations regarding chromium(III) (Hong et al. 2005). Higher concentration of some metal cations such as lanthanum(III), iron(II,III), and lead(II) between 100 µM and 1 mM activated PTP1B.

**Table 3:** Effects of metal cations and oxoanions on PTP1B activity

Effector cation/oxyanion	Nature of modulation I:inhibition A:activation	Concentration with significant modulation	Modulation (%)
Zinc (Zn^2+^)	I	10 μM	95
Cadmium (Cd^2+^)	I	10 μM	91
Copper (Cu^2+^)	I	5 μM	99
Lead (Pb^2+^)	I/A	5 μM/1 mM	96/87
Silver (Ag^+^)	I	50 μM	92
Iron (Fe^2+^)	I/A	5 μM/500 μM	77/74
Iron (Fe^3+^)	I/A	10 μM/500 μM	71/68
Chromium (Cr^3+^)	NS^a^	–	–
Lanthanum (La^3+^)	I/A	1 μM/100 μM	63/15
Lithium (Li^+^)	NS^a^	–	–
Manganese (Mn^2+^)	NS^a^	–	–
Nickel (Ni^2+^)	NS^a^	–	–
Tetrathiomolybdate (MoS_4_^2−^)	I	100 μM	97
Tungstate (WO_4_^2−^)	I	500 μM	62
Nitrate (NO_3_^−^)	I	100 mM	65
Heptamolybdate (Mo_7_O_24_^6−^)	I	100 μM	88
Arsenate (AsO_4_^3−^)	I	500 μM	66
Chromate (CrO_4_^2−^)	I	500 μM	81
Borate (BO_3_^3−^)	NS^a^	–	–
Carbonate (CO_3_^2−^)	NS^a^	–	–

^a^ *NS* no significant effect. n = 3

### Femtomolar and nanomolar concentrations of Cu^2+^, Cd^2+^ and Zn^2+^ inhibit PTP1B

**Fig. 1:**
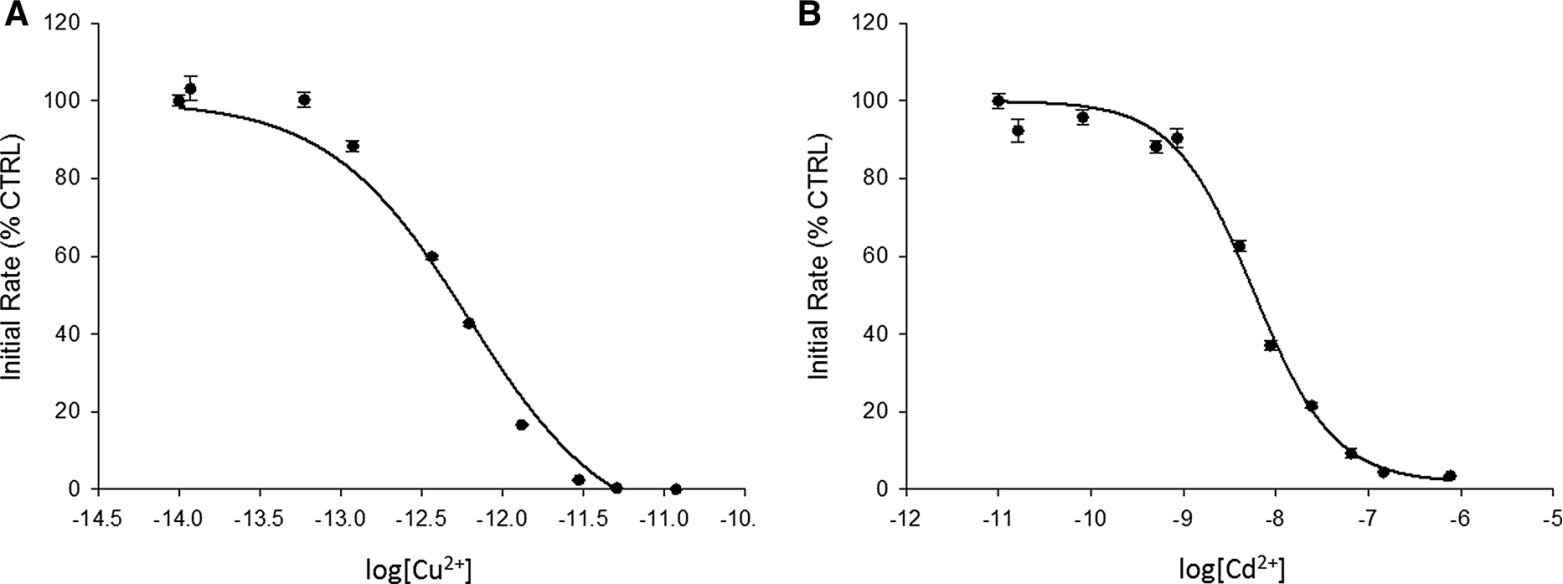
Cu^2+^ and Cd^2+^ inhibition of PTP1B. The enzyme was assayed with increasing concentrations of free copper(II) ions (**a**) and cadmium(II) ions (**b**) as calculated using Maxchelator (Table 1) in a buffer containing 50 mM Hepes, 1 mM NTA, 0.1 mM TCEP and 0.01% (v/v) Triton X-100, pH 7.4. Experiments were performed in triplicate for each metal ion. The enzyme was added to the buffer containing the metal cations and 3 µM DiFMUP

**Fig. 2:**
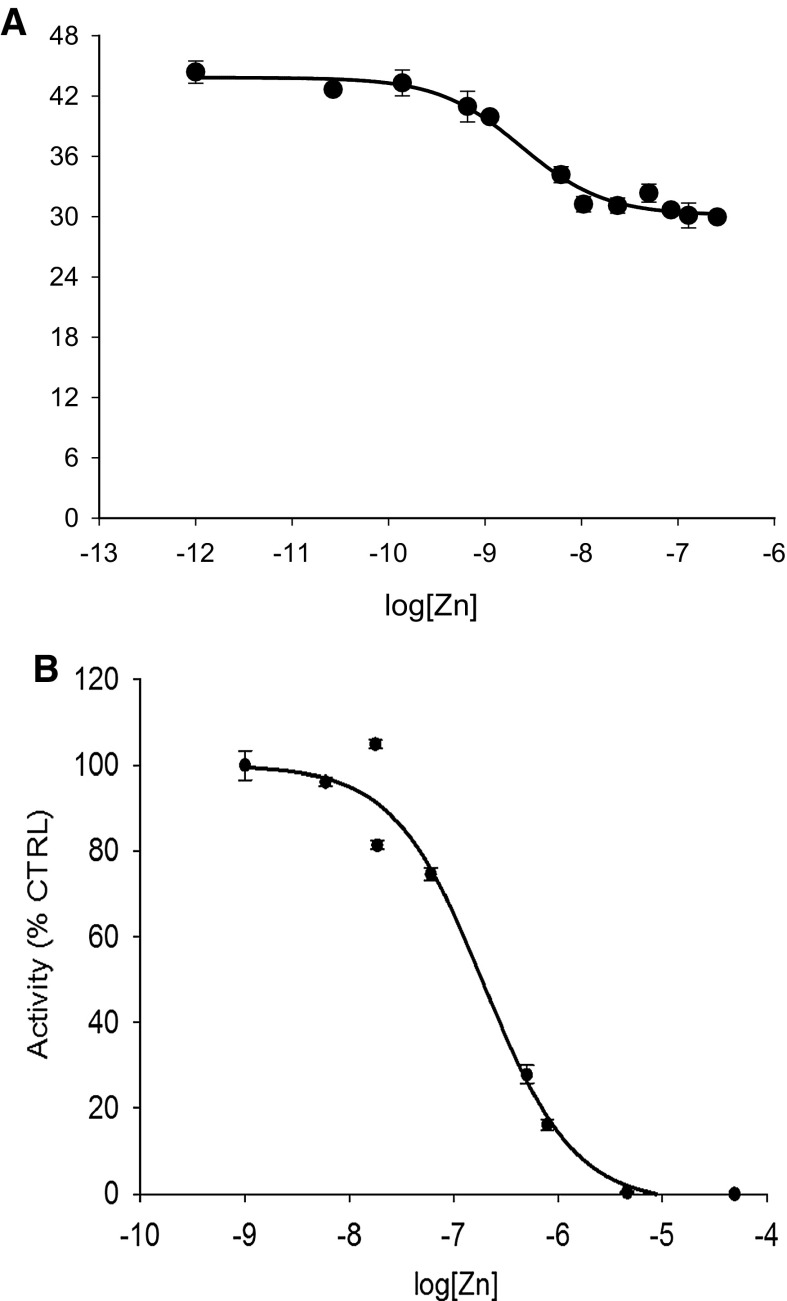
Zn^2+^ inhibition of PTP1B in the absence and presence of glutathione. The enzyme was assayed with increasing concentrations of free zinc(II) ions as calculated using Maxchelator (Table 1) in a buffer containing 50 mM Hepes, 1 mM NTA, 0.1 mM TCEP and 0.01% (v/v) Triton X-100, pH 7.4 (A) or in a buffer containing 50 mM Hepes, pH 7.4, 1 mM GSH and 0.01% (v/v) Triton X-100 (B). The enzyme was assayed with increasing concentrations of free zinc (II) ions according to calculations provided by Professor Wojciech Bal (Polish Academy of Science, Warsaw) (Table 2). Experiments were performed in triplicate. Enzyme was added to the buffer containing zinc(II) ions and 3 µM DiFMUP

In addition to Zn^2**+**^, we determined the inhibition for Cu^2+^ and Cd^2+^, two cations with high affinity to ligands according to the Irving-Williams series. Based on free Cu^2+^, Cd^2+^ and Zn^2**+**^ concentrations and fitting the data to a non-linear regression curve, we obtained inhibition constants of 600 fM, 7.3 nM and 2.4 nM, respectively (Figs. 1a, b and 2a). In contrast to Cu^2+^ and Cd^2+^, Zn^2**+**^ did not inhibit PTP activity completely under these conditions (Fig. 2a).

### Effect of Zn^2+^ buffered with glutathione on PTP1B activity

Glutathione (GSH) serves as a redox and metal buffer in cells. When using 1 mM GSH instead of 1 mM NTA and 0.1 mM TCEP in the assay solution, Zn^2+^ inhibited the enzyme completely with an inhibition constant of 200 nM (Fig. 2b).

### Effect of oxyanions on PTP1B activity: inhibition constants of vanadate, molybdate, tungstate, arsenate, and nitrate

Some metal oxyanions are analogues of phosphate, which is the hydrolytic product of the PTP reaction. Phosphate is a competitive inhibitor of PTP1B with a *K*_i_ value of 17 mM (Zhang and Zhang 1998). Other oxyanions with structural similarities to phosphate also inhibit PTPs, e.g. vanadate, molybdate, tungstate and arsenate. Among them, vanadate seems to be the strongest inhibitor of PTP1B with a *K*_i_ value of 0.38 µM (Huyer et al. 1997). Among the metal oxyanions we investigated, tetrathiomolybdate and heptamolybdate showed the strongest inhibition (97 and 88% respectively) at a concentration of 100 µM. Tungstate, arsenate and chromate inhibited PTP1B activity 62–81% at higher concentrations between 500 µM and 100 mM (Table 3). Among the non-metal oxyanions tested, nitrate inhibited a bit weaker than phosphate, while borate and carbonate did not inhibit significantly.

**Fig. 3:**
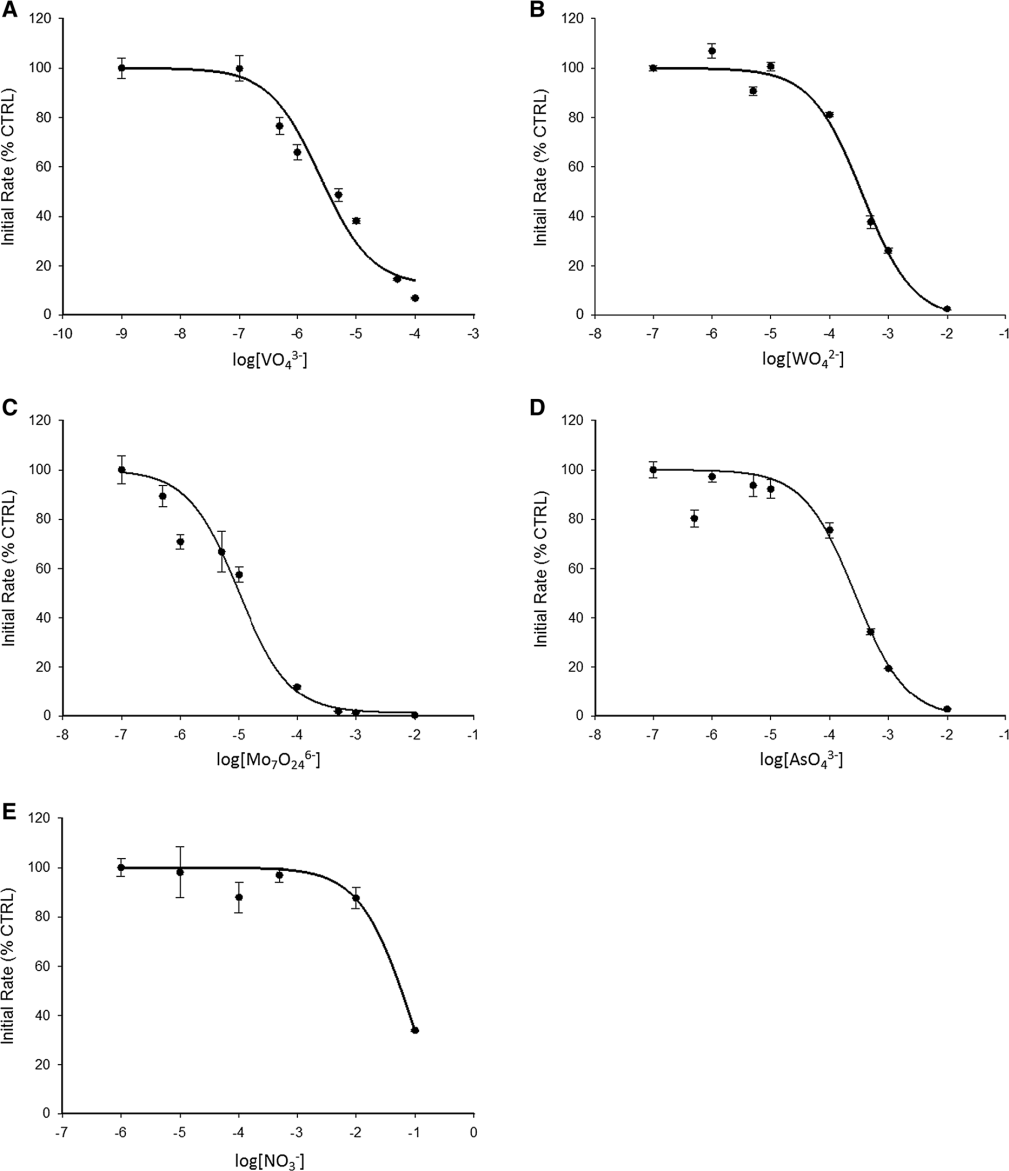
Oxyanions inhibit PTP1B. The enzyme was assayed with increasing concentrations of vanadate (**a**), tungstate (**b**), heptamolybdate (**c**), arsenate (**d**) and nitrate (**e**) in a buffer containing 50 mM Hepes, 1 mM NTA, 0.1 mM TCEP and 0.01% (v/v) Triton X-100, pH 7.4. Experiments were performed in triplicate for each oxyanion. The enzyme was added to the buffer containing the oxyanions and 3 µM DiFMUP

Under identical assay conditions, the apparent *K*_i_ (IC_50_) values are 1.5 µM (vanadate), 9 µM (heptamolybdate), 200 µM (molybdate) (data collected by Ms. Sherry Sachdeva), 210 µM (tungstate), 200 µM (arsenate), and 54 mM (nitrate) (Fig. 3a–e). The different inhibition constants may reflect different ionization states of the anion at the pH of investigation, in the case of vanadate the propensity to form a covalent intermediate analogous to the phospho-intermediate in contrast to the other anions that form Michaelis-like complexes, and last but not least geometric factors, such as in the case of nitrate, which has a planar geometry.

## Discussion

### Inhibition by oxyanions

In an overview of the molecular and ionic mimicry of metal ions, it was summarized that arsenate (As(V)) and vanadate (V(V)) are structurally similar to endogenous phosphate (P(V)) and mono-anions due to partial ionization while chromate (Cr(VI)) and molybdate (Mo(VI)) are structurally similar to endogenous sulphate (S(VI)) are fully ionized and hence di-anions (Clarkson 1993). Metal oxyanions that are analogues of phosphate inhibit PTP1B. Vanadate is a transition state inhibitor forming a covalent intermediate with the active site cysteine of the enzyme (Brandao et al. 2010; McLauchlan et al. 2015). Molybdate and tungstate are product inhibitors and do not form covalent intermediates. Oxyanions of non-metals also bind at the active site and inhibit the enzyme, i.e. sulphate and sulphonates such as Hepes buffer, arsenate, and nitrate. The protonation state of the anion, i.e. the p*K*_a_ value of its conjugate acid, is important for inhibition. For example, in the case of phosphate, the dianion binds. Hydrogenphosphate (HPO_4_^2−^) interacts with the guanidinium group of Arg221 of PTP1B in its closed conformation. For phosphoric acid the p*K*_a_ values are 2.0, 5.7 and 11.7 while they are 3.8, 7.8, and 13 for H_3_VO_4_ (25 °C, I = 0.5). The stereochemistry is also important. In a crystal structure of PTP1B in the presence of nitrate, nitrate is bound in the active site and 50% of the protein molecules are in the closed conformation (Kenny et al. 2014). Inhibition constants were not reported, however. We show that nitrate, which is a monoanion and has a planar geometry, indeed inhibits PTP1B but rather weakly with an IC_50_ value of 54 mM. The inhibition is expected to be stronger in the closed conformation of the enzyme because additional interactions of nitrate with the side chains of Asp181 and Arg221 were observed (Kenny et al. 2014).

The inhibition constants for molybdate and tungstate are similar. There is no significant difference between the p*K*_a_ values (20 °C, I = 0.1) of molybdic acid (p*K*_a1_ = 4.2; p*K*_a2_ = 8.2) and tungstic acid (p*K*_a1_ = 3.5; p*K*_a2_ = 8.1) (Smith and Martell 1976). However, the inhibition of PTP1B by heptamolybdate is about one order of magnitude stronger. An investigation of Keggin compounds that hydrolyse to the mononuclear anions in water also shows that molybdate and tungstate inhibit with virtually the same IC_50_ values (21 and 25 µM respectively) at pH 7.5 (Heo et al. 2002). Yet, there are structural differences. The crystal structure of PTP1B in the presence of phosphomolybdate shows the MoO_3_ moiety (derived from phosphomolybdate by hydrolysis) binding at the active site. The molybdenum atom is six-coordinate with three oxo-ligands in MoO_3_, two apical water molecules and an S atom from the catalytic cysteine residue, while in the complex with tungstate the WO_4_ moiety is five-coordinate with four oxo-ligands and an interaction with the S atom of the catalytic cysteine. In contrast to the molybdate complex, the side chain of Gln262 of PTP1B interacts with one of the oxo-ligands in the tungstate complex (Heo et al. 2002). Unlike in the complex with vanadate the protein is in the open conformation and there is no interaction with the side chain of Arg221. The crystal structure of PTP1B with tungstate shows the tetrahedral anion bound only to NH groups of the peptide backbone in the open conformation of the protein (Barford et al. 1998). However, the Yersinia PTP complex with tungstate is in the closed conformation with additional interactions with the active site Asp, Arg, and Gln residues (Fauman et al. 1996). The crystal structure of bovine low molecular weight PTP with molybdate reveals that the molybdenum atom is coordinated with six ligands: three oxo-ligands, two apical water molecules and an S atom of the catalytic cysteine residue (Zhang et al. 1997). For this enzyme, the authors report inhibition constants of 9 and 210 µM for molybdate and tungstate, respectively. Thus, a major issue remains whether or not measured inhibition constants relate to the open or to the closed conformation of the enzyme. Additional stabilization in the closed conformation suggests tighter binding. In order to further elucidate the inhibition properties we investigated the third member of this group, namely chromate. It is a weaker inhibitor than molybdate with an IC_50_ value of about 400 µM. Chromic acid is a much stronger acid than either molybdic acid or tungstic acid (p*K*_a_ = −0.98 for H_2_CrO_4_). Moreover, chromate is a larger oxyanion than molybdate (Bridges and Zalups 2010).

Arsenate inhibits PTP1B with a *K*_i_ of 150 µM (pH 7.0) (Zhang and Zhang 1998). Under our experimental conditions, arsenate inhibits PTP1B with similar strength (IC_50_ ≈ 200 μM, pH 7.4). The p*K*_a2_ of arsenate is 7.03–7.10 and thus between those of phosphate and vanadate. A crystal structure of PTP1B with arsenate has not been reported. However, several crystal structures of arsenate reductase have been solved and shown that the structure of this enzyme is related to the PTP family (Hu et al. 2015). Remarkably, the protein phosphatase CD45 belonging to the PTP superfamily reduces arsenate. A number of eukaryotic enzymes that function as arsenate reductases are homologues of the catalytic domain of the human Cdc25 phosphatase, suggesting that Cdc25 has the potential to reduce arsenate to the more toxic arsenite. It may provide a framework to identify other human PTPs containing active sites that might moonlight as arsenate reductases (Bhattacharjee et al. 2010).

Other oxyanions like borate show no significant effect on PTP1B activity. Borate is a monoanion, such as nitrate and hydrogencarbonate, with a p*K*_a_ value of 9.23 for the transition B(OH)_3_ to H_2_BO^−^. Hence it is expected to interact weakly with PTPs.

### Inhibition by cations

Inhibition of PTP1B by zinc (II) ions has been known for a long time (Brautigan et al. 1981). However, it was not known where zinc binds, inhibition constants were not obtained, and the inhibition was not discussed in the context of the available free zinc ion concentrations in the cell. It is now known that fluctuating zinc ion concentrations in the cell have signalling functions and a significant body of work indicates an effect on phosphorylation signalling (Maret 2009; Bellomo et al. 2016). The inhibition is much stronger than originally reported (21 pM for RPTPβ/PTPRB (Wilson et al. 2012) and 5–15 nM for PTP1B (Haase and Maret 2003; Krezel and Maret 2008). The strong binding is thought to be due to zinc binding in the closed conformation of the enzyme when additional interactions with the phosphocysteinyl residue and the catalytic aspartate are possible (Bellomo et al. 2014). The investigations established zinc ions as a physiological modulator of protein tyrosine phosphatases with wide implications for signal transduction (Bellomo et al. 2016). In the present investigation, we addressed the role of other metal ions, which unlike calcium and zinc change concentrations only under pathological conditions and under environmental (nutritional, pharmacological, toxicological) exposure. Cellular metal ions are buffered and the free metal ion concentrations depend on the buffering capacity. Thus, cellular metal ion concentrations are not just influenced by the metal ion concentrations available extracellularly but also by any factor that changes the buffering capacity of the cell. Whether reactions are physiologically significant depends on this buffering capacity. Outside this range of the cellular metal ion buffering capacity, metals have pharmacological or toxicological actions. As with anions charge is perhaps the most important factor for divalent metal ions inhibiting PTPs. With the exception of thiophilic cations such a silver(I) ions, mono- and trivalent metal ions inhibit PTP1B relatively weakly. Since the strength of binding of divalent cations generally follows the Irving-Williams series, we focused on two other strongly binding divalent cations in addition to zinc, namely cadmium and copper.

### Cadmium

Cd^2+^ binds as strong as Zn^2+^, but in contrast to zinc the interaction is complete without incubating the protein with the metal ion, suggesting that cadmium, which has a higher affinity to sulphur than zinc, inhibits the enzyme in the open conformation. We noted that zinc in the presence of glutathione also gives complete inhibition. Glutathione (GSH) is both a reducing and a complexing agent and therefore NTA and TCEP are not required when assaying PTP1B. Glutathione forms complexes with zinc, including ternary complexes with ligands such as histidine (Krezel et al. 2003). Hence glutathione and zinc may bind together in the open conformation of the enzyme.

### Copper

Cu^2+^ is the most potent inhibitor of PTP1B with an IC_50_ value of 0.6 pM. As copper (II) ions oxidize the thiol of cysteine in the presence of oxygen, they are irreversible inhibitors. Thus, the highly potent Cu^2+^ inactivation of the PTPT VHR was discussed as a consequence of the cupric ions oxidizing the active-site cysteine (Zhu et al. 2013). In the cell, copper is mostly in the form of copper(I) and therefore it is important to investigate copper(I) complexes. They inhibit PTPs more weakly (Wang et al. 2010, 2011). Exposure of cells to copper(II) complexes results in inhibition of PTPs. The proposed mechanism of inhibition of PTP1B by the copper(II) complex (bis-thiosemicarbazonato copper complexes) involves the complex entering the cell, Cu^2+^ reduction to Cu^+^ in the cell, and finally inhibition of PTP1B activity, leading, for example, to sustained phosphorylation and activation of the epidermal growth factor receptor (Price et al. 2009).

Inhibition of PTPs by metal ions and metal ion complexes was investigated extensively by others (Table 4). However, our investigations demonstrate that interpretation and comparison of data is hampered by many factors. In addition to redox, metal ion, and pH control in assays, interactions between cations and anions need to be considered. A synergism or antagonism would make the binding constant of one species dependent on that of the other. Thus, anions and cations of the same element in different oxidation may bind, i.e. the vanadate (V(V)) anion and the oxyvanadium (V(IV)) cation or Cr(III)/Cr(VI)(chromate). Zinc concentrations need to be considered and controlled when testing other anions or cations because zinc is a common contaminant of many reagents at concentrations causing strong inhibition of PTPs. The inhibition constants need to be interpreted in terms of the species of the inhibitor and the form of the enzyme inhibited. For example, arsenate binds at the anion binding site in the closed conformation of the enzyme whereas As(III) compounds such as the arsenicals monomethylarsonous acid and dimethylarsinous acid have high affinity to the sulphhydryl groups of PTP1B and lead to irreversible inhibition (Kanwal et al. 2012). Depending on the affinity of the cation for sulphur, the metal ion can bind to the protein in the open conformation and/or the closed conformation. If it binds to both forms, the measured inhibition constant will be the product of two different molecular constants. Once the binding constants are obtained under defined conditions, they need to be interpreted in terms of the metal ion availability in the cell to assign physiological, pharmacological, or toxicological importance. Such a consideration strengthens or weakens the argument for a cation or an anion being important for the inhibition of the enzyme(s) in vivo. Strictly, we show these effects for PTP1B only but the inhibition observed for many other PTPs (Table 4) make our results more generally applicable.

**Table 4:** Metal ions/metal ion complexes inhibiting human protein tyrosine phosphatases

Metal ion/metal ion complex	PTP1B	SHP-1	SHP-2	TCPTP	Reference
Zinc	3–17 nM	93 nM	1–2 µM	200 nM	Bellomo et al. (2014); Haase and Maret (2005); Haase and Maret (2003)
Copper	0.14 µM	0.18 µM	–	0.15 µM	Zhu et al. (2013)
Vanadate	0.38–33 µM	13 µM	–	–	Huyer et al. (1997); Heo et al. (2002)
Tungstate	20–210 µM	–	–	–	Zhang et al. (1997); Heo et al. (2002)
Molybdate	9–3 µM	10–20 µM	–	–	Zhang et al. (1997); Heo et al. (2002)
Arsenate	4.3 mM	–	–	–	Zhang and Zhang (1998)
Dinuclear copper complex	0.15 µM	0.23 µM	>100 µM	1.81 µM	Ma et al. (2011)
Mononuclear iron dicitrate	–	250 µM	–	–	Gomez et al. (2010)
Vanadium complex	0.03–1 µM	–	–	–	Lu and Zhu (2014)
Bis-(maltolato)oxovanadium(IV)	0.86 µM	–	–	–	Li et al. (2008)
Sodium stibogluconate	100 µg/ml	10 µg/ml	100 µg/ml	–	Lu and Zhu (2011)

## Conclusions

We conclude that a variety of metal cations and metal oxyanions are rather potent PTP1B inhibitors while non-metal oxyanions are generally weak inhibitors. Among cations, Cu^2+^ ions inhibit PTP1B activity at femtomolar concentrations while Cd^2+^ and Zn^2+^ ions inhibit at nanomolar concentrations. Glutathione affects the mode of zinc inhibition of PTP1B activity. The inhibition is thought to occur in the enzyme in the open conformation (for highly thiophilic metal ions) and/or in the closed conformation of the enzyme (for less thiophilic metal ions). Investigations at varying pH values, lack of knowledge of the type of inhibition and control of anions and cations make it difficult or nearly impossible to compare inhibition data of PTPs in the literature. PTP1B has been and continues to be a major therapeutic target for inhibition by low molecular weight compounds and drug candidates (Heneberg 2009) because of its role in many important physiological and pathophysiological processes (diabetes, cancer, neurodegeneration). Understanding the structural basis of the interactions of PTP1B with metal ions/metal ion complexes and possible synergism between cations and anions will be important for future design of novel therapeutic agents and for addressing the molecular toxicology of metal ions and the cumulative risks associated with exposures.

## Abbreviations

PTP: Protein tyrosine phosphatase
GSH: Glutathione

## References

[CR1] Barford D, Das AK, Egloff MP (1998). The structure and mechanism of protein phosphatases: insights into catalysis and regulation. Annu Rev Biophys Biomol Struct.

[CR2] Bellomo E, Massarotti A, Hogstrand C, Maret W (2014). Zinc ions modulate protein tyrosine phosphatase 1B activity. Metallomics.

[CR3] Bellomo E, Singh KB, Massarotti A, Hogstrand C, Maret W (2016). The metal face of protein tyrosine phosphatase 1B. Coord Chem Rev.

[CR4] Bers DM, Patton CW, Nuccitelli R (2010). A practical guide to the preparation of Ca^2+^ buffers. Methods Cell Biol.

[CR5] Bhattacharjee H, Sheng J, Ajees AA, Mukhopadhyay R, Rosen BP (2010). Adventitious arsenate reductase activity of the catalytic domain of the human Cdc25B and Cdc25C phosphatases. Biochemistry.

[CR6] Brandao TA, Hengge AC, Johnson SJ (2010). Insights into the reaction of protein-tyrosine phosphatase 1B: crystal structures for transition state analogs of both catalytic steps. J Biol Chem.

[CR7] Brautigan DL, Bornstein P, Gallis B (1981). Phosphotyrosylprotein phosphatase. Specific inhibition by Zn. J Biol Chem.

[CR8] Bridges CC, Zalups RK, Zalups RK, Koropatnick J (2010). Ionic and molecular mimicry and the transport of metals. Cellular and molecular biology of metals.

[CR9] Clarkson TW (1993). Molecular and ionic mimicry of toxic metals. Annu Rev Pharmacol Toxicol.

[CR10] Fauman EB, Yuvaniyama C, Schubert HL, Stuckey JA, Saper MA (1996). The X-ray crystal structures of Yersinia tyrosine phosphatase with bound tungstate and nitrate. Mechanistic implications. J Biol Chem.

[CR11] Gomez MA, Alisaraie L, Shio MT, Berghuis AM, Lebrun C, Gautier LI, Olivier M (2010). Protein tyrosine phosphatases are regulated by mononuclear iron dicitrate. J Biol Chem.

[CR12] Haase H, Maret W (2003). Intracellular zinc fluctuations modulate protein tyrosine phosphatase activity in insulin/insulin-like growth factor-1 signaling. Exp Cell Res.

[CR13] Haase H, Maret W (2005). Fluctuations of cellular, available zinc modulate insulin signalling via inhibition of protein tyrosine phosphatases. J Trace Elem Med Biol.

[CR14] Heneberg P (2009). Use of protein tyrosine phosphatase inhibitors as promising targeted therapeutic drugs. Curr Med Chem.

[CR15] Heo Y-S, Ryu JM, Park SM, Park JH, Lee H-C, Hwang KY, Kim J (2002). Structural basis for inhibition of protein tyrosine phosphatases by Keggin compounds phosphomolybdate and phosphotungstate. Exp Mol Med.

[CR16] Hong W, Allison K, Brautigan DL (2005). Cellular chromium enhances activation of insulin receptor kinase. Biochemistry.

[CR17] Hu C, Yu C, Liu Y, Hou X, Liu X, Hu Y, Jin C (2015). A hybrid mechanism for the Synechocystis arsenate reductase revealed by structural snapshots during arsenate reduction. J Biol Chem.

[CR18] Huyer G, Liu S, Kelly J, Moffat J, Payette P, Kennedy B, Tsaprailis G, Gresser MJ, Ramachandran C (1997). Mechanism of inhibition of protein-tyrosine phosphatases by vanadate and pervanadate. J Biol Chem.

[CR19] Kanwal R, Zhe C, Wen WW, Yan WW, Akira S, Yan FZ, Hua N, Noriyuki S (2012). Mechanisms underlying the inhibitory effects of arsenic compounds on protein tyrosine phosphatase (PTP). Toxicol Appl Pharmacol.

[CR20] Kenny PW, Newman J, Peat TS (2014). Nitrate in the active site of protein tyrosine phosphatase 1B is a putative mimetic of the transition state. Acta Cryst D.

[CR21] Krężel A, Maret W (2008). Thionein/metallothionein control Zn(II) availability and the activity of enzymes. J Biol Inorg Chem.

[CR22] Krężel A, Wójcik J, Maciejczyk M, Bal W (2003). May GSH and L-His contribute to intracellular binding of zinc? Thermodynamic and solution structural study of a ternary complex. Chem Commun.

[CR23] Li M, Ding W, Baruah B, Crans DC, Wang R (2008). Inhibition of protein tyrosine phosphatase 1B and alkaline phosphatase by bis(maltolato)oxovanadium (IV). J Inorg Biochem.

[CR24] Lu L, Zhu M (2011). Metal-based inhibitors of protein tyrosine phosphatases. Anti-Cancer Agents Med Chem.

[CR25] Lu L, Zhu M (2014). Protein tyrosine phosphatase inhibition by metals and metal complexes. Antiox Redox Signal.

[CR26] Ma L, Lu L, Zhu M, Wang Q, Gao F, Yuan C, Wu Y, Xing S, Fu X, Mei Y, Gao X (2011). Dinuclear copper complexes of organic claw: potent inhibition of protein tyrosine phosphatases. J Inorg Biochem.

[CR27] Maret W (2009). Molecular aspects of human cellular zinc homeostasis: redox control of zinc potentials and zinc signals. Biometals.

[CR28] Maret W (2013). Inhibitory zinc sites in enzymes. Biometals.

[CR29] Smith RM, Martell, AE (1976) Critical stability constants, vol. 4. Inorganic complexes. Springer, New York

[CR30] McLauchlan CC, Peters BJ, Willsky GR, Crans DC (2015). Vanadium-phosphatase complexes: phosphatase inhibitors favor the trigonal bipyramidal transition state geometries. Coord Chem Rev.

[CR31] Price KA, Caragounis A, Paterson BM, Filiz G, Volitakis I, Masters CL, Barnham KJ, Donnelly PS, Crouch PJ, White AR (2009). Sustained activation of glial cell epidermal growth factor receptor by bis(thiosemicarbazonato) metal complexes is associated with inhibition of protein tyrosine phosphatase activity. J Med Chem.

[CR32] Tonks NK (2013). Protein tyrosine phosphatases-from housekeeping enzymes to master regulators of signal transduction. FEBS J.

[CR33] Wang Q, Lu L, Yuan C, Pei K, Liu Z, Guo M, Zhu M (2010). Potent inhibition of protein tyrosine phosphatase 1B by copper complexes: implications for copper toxicity in biological systems. Chem Commun.

[CR34] Wang Q, Zhu M, Lu L, Yuan C, Xing S, Fu X (2011). Potent inhibition of protein tyrosine phosphatases by quinquedentate binuclear copper complexes: synthesis, characterization and biological activities. Dalton Trans.

[CR35] Wilson M, Hogstrand C, Maret W (2012). Picomolar concentrations of free zinc (II) ions regulate receptor protein tyrosine phosphatase β activity. J Biol Chem.

[CR36] Zhang YL, Zhang ZY (1998). Low-affinity binding determined by titration calorimetry using a high-affinity coupling ligand: a thermodynamic study of ligand binding to protein tyrosine phosphatase 1B. Anal Biochem.

[CR37] Zhang M, Zhou M, Van Etten RL, Stauffacher CV (1997). Crystal structure of bovine low molecular weight phosphotyrosyl phosphatase complexed with the transition state analog vanadate. Biochemistry.

[CR38] Zhu R, Lu L, Zhu M, Han H, Yuan C, Xing S, Fu X (2013). Synthesis and evaluation of copper complexes of Schiffbase condensates from 5-substituted-2-hydroxybenzaldehyde and 2-substituted-benzenamine as selective inhibitors of protein tyrosine phosphatases. Inorg Chim Acta.

